# Haplotype-based validation of genomic regions for breeding of new Nordic apples

**DOI:** 10.1186/s41065-026-00728-0

**Published:** 2026-08-12

**Authors:** Jonas Skytte af Sätra, Dag Røen, Tuuli Haikonen, Kimmo Rumpunen, Larisa Garkava-Gustavsson, Stein Harald Hjeltnes

**Affiliations:** 1 Department of Plant Breeding, Swedish University of Agricultural Sciences, Alnarp, Sweden; 2 Graminor / Njøs Fruit and Berry Centre, Leikanger, Norway; 3 Natural Resources Institute Finland (Luke), Production Systems, Toivonlinnantie 518, Piikkiö, FI-21500 Finland

**Keywords:** Validation, Haplotype, *Malus domestica*, Harvest date, Flowering date, Polyphenols

## Abstract

When breeding apple cultivars for northern latitudes, early harvest and late flowering are desirable to manage the short growing season and ongoing climatic changes. Depending on the intended use, increased content of polyphenolic compound might be considered desirable (juice and cider) or undesirable (dessert). Thus, we phenotyped a total of 111, 189, and 88 apple genotypes for harvest date, flowering date, and peel concentration of procyanidin B2 (PRB2), respectively, across three Nordic locations in Sweden, Norway, and Finland. Using phased marker data from the 20 K apple Infinium^®^ SNP array we performed haplotype-based validation of previously described quantitative trait loci (QTL) intervals and genomic regions on Linkage Group (LG) 3 (harvest date), LG 9 and 12 (flowering), and LG16 (PRB2). We assessed the correlation between the traits and historical climate adaptation and identified haplotypes associated with increasing and decreasing effects for the three traits. These results are valuable for Marker Assisted Parent Selection in the Nordic breeding programs, as they support designing crosses to meet the demands of current and future climatic conditions, and fruit quality towards breeding of new dessert cultivars, or cultivars for juice or cider production.

**Supplementary Information:**

The online version contains supplementary material available at 10.1186/s41065-026-00728-0.

## Introduction

Climatic adaptation of woody perennial plant species is important and is expressed through different phenological traits. In apple (*Malus domestica* Borkh.) harvest date is an important adaptive trait for climatic conditions. At high latitudes with short growing periods, early maturing cultivars are required as late maturing cultivars are unlikely to reach an acceptable maturity stage. Similarly, time of flowering has emerged as an important breeding target in order to avoid extensive spring frost damages under a changing climate [1–4]. Fruit concentration of polyphenolic compounds is not expected to be related to climate adaptation but might constitute an important breeding target. Polyphenolic compounds such as flavanols give rise to astringency and bitterness of apple fruit and juice. Thus, for breeding of dessert apples low concentrations might be favorable, while in breeding of cultivars for juice and cider production high concentrations might be preferred. Both flowering and harvest date require extensive phenotyping over several years to get reliable phenotypic data. Thus, genomic tools to facilitate marker assisted parent selection (MAPS) or marker assisted seedling selection (MASS), or both, would be valuable for applications in Scandinavian breeding programs. The same is valid for content and composition of phenolic compounds.

The previous decade was marked by major developments in genomic tools for genetic studies in apple, including SNP arrays [5–7], Genotyping-By-Sequencing [8], the iGLMap [9], several reference whole genome sequences (WGSs) [10–14], FlexQTL™ for pedigree-based-analysis (PBA) [15, 16], and pipelines for generation of highly curated phased marker datasets [17]. These tools were successfully used for genetic dissection of traits and identification of marker-trait associations through Genome Wide Association Studies (GWAS) and quantitative trait loci (QTL) discovery, primarily by PBA through multiparental populations (MPPs). In GWAS, marker-trait associations for the trait of interest are identified by phenotyping a screening panel, consisting of preferably unrelated individuals and genotyped with a preferably unbiased high-density marker system. QTL discovery is conducted by phenotyping of one or several full-sibling (FS) families, which are genotyped with a marker system that is usually of lower density than for GWAS. The data is then used in linkage analysis in a single family or PBA analysis in multiple families connected through their pedigrees. However, prior to deployment for marker assisted selection (MAS) in a specific breeding germplasm, genomic regions and QTL intervals should be validated in the specific genetic background and under relevant environmental conditions [18].

Recent achievements in the genetic dissection of adaptive traits open new possibilities for breeding apple cultivars better suited for production at high latitudes, including diverse uses such as juice and cider. For example, loci around *NAC18.1* on chromosome (Chr) 3 have been found to be strongly associated with harvest date [19–22], making it a promising target for breeding for early maturing cultivars for northern Scandinavia. A number of QTL intervals have been found to segregate for flowering date [23], with two QTL on LG9 and 12 having very strong statistical support for regulation of bud break expressed in both calendar-days and day-degrees. Lastly, genomic regions and QTL intervals around the *LAR1* locus on LG16 have been identified in different apple germplasms [24, 25]. This locus encodes the enzyme leucoanthocyanidin reductase (LAR), which catalyzes the conversion of leucocyanidin to the flavanol catechin in the phenylpropanoid pathway [26]. Previous studies have reported peel concentration of flavanols to be highly correlated among themselves, and with flesh concentration of flavanols [25]. The *LAR1* locus appears to affect procyanidin content in both peel and flesh of apple fruits and Khan et al. [27] reported markers around the *LAR1* locus to segregate for all reported flavanols, in both peel and flesh of the fruit. In this work, we focus on the flavanol procyanidin B2 (PRB2), an epicatechin dimer and a major phenolic compound in apple that is important for astringency and bitterness.

The aim of the current study was to provide information which will enable MAS in Nordic apple breeding programs, considering harvest date, time of full bloom, and concentration of PRB2. Thus, the objectives were to (i) assess the relation between the three traits and historical climate adaptation across three Nordic locations in Sweden, Norway, and Finland, (ii) validate the effect of previously published genomic regions and QTL intervals for harvest date on Chr3, flowering date on LG 9 and 12, and PRB2 concentration on LG16. Then, (iii) identify haplotypes associated with contrasting trait effects at each locus and estimate their effect sizes.

## Materials and methods

### Plant material

**Fig. 1:**
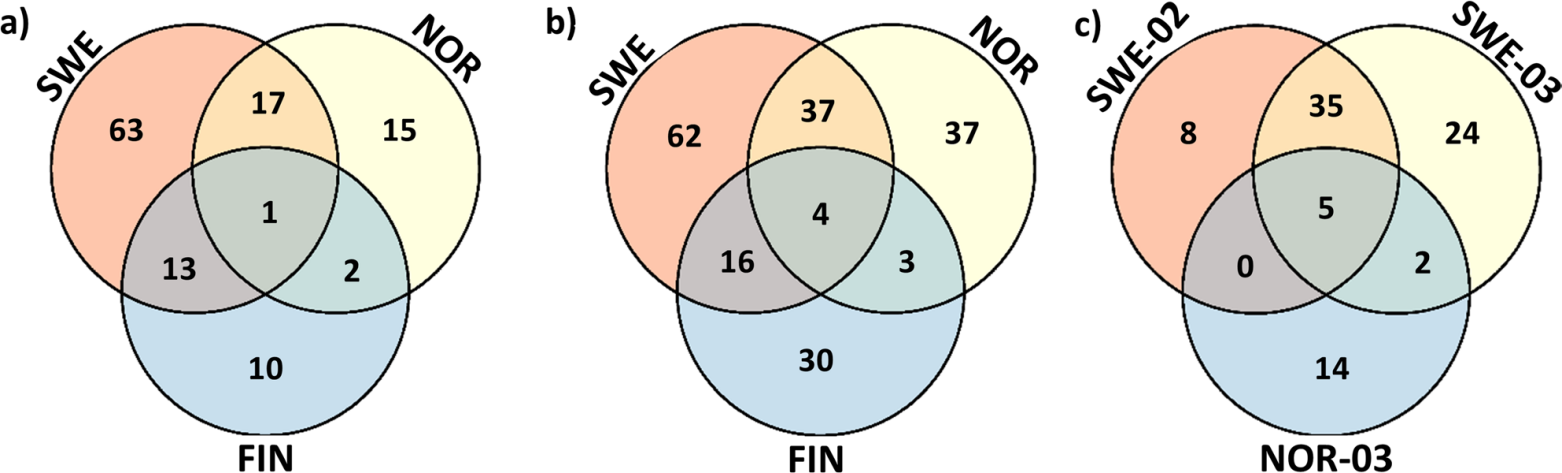
Connectivity between phenotypic data sets from Sweden, Norway, and Finland for harvest date (**a**), flowering date (**b**), and peel concentration of procyanidin B2 (PRB2) (**c**). Note that for c), the different years of Swedish phenotyping are indicated

A total of 189 unique diploid apple genotypes were phenotyped for harvest date or flowering date, or both traits, in Sweden (total 123 genotypes, Balsgård, Kristianstad, 56.1 ddN), Norway (total 81 genotypes, Graminor/Njøs Fruit and Berry Centre [NJØS], Leikanger, 61.2 ddN), and Finland (total 53 genotypes, Luke, Piikkiö, 60.4 ddN) (Fig. 1, Supplementary Table 1). For harvest date the only cultivar in common between the three locations was ‘Wealthy’, while for flowering date the cultivars ‘Lobo’, ‘Oranie’, ‘Wealthy’, and ‘Åkerö’ were common between the three locations.

In the apple collections at Balsgård two trees of each genotype were used for phenotyping. The majority of trees were grafted on the semi-dwarfing rootstock ‘M.26’, planted from 2003 to 2016.

The apple cultivar collection of Graminor / NJØS was planted 2014–2016 with 3 trees per cultivar grafted mainly on rootstock ‘M.9’, but some weak-growing cultivars were grafted on rootstock ‘B.9’.

In the Finnish apple collection in Piikkiö, Kaarina, on average two trees of each genotype (1–10 trees for flowering time and 1–3 trees for harvest time) were used for phenotyping. The majority of trees were grafted on the vigorous clonal rootstock ‘YP’ and planted in 2013 or were own-rooted established from micro-shoot cultures and planted as large plants in 2007 [28, 29]. In all locations, the trees were annually pruned and cultivated using Integrated Production (IP) practices.

Data on air temperatures for calculations of day-degrees in 2019, 2020, and 2021 were collected from the Kristianstad weather station (Swedish Meteorological and Hydrological Institute, Sweden), the Njøs weather station (Agrometeorology Norway, lmt.nibio.no), and the Kaarina Yltöinen weather station (Finnish Meteorological Institute, Finland). Historical data on the highest recommended growing-zone (HGZ) was retrieved from Swedish pomological records, primarily Nilsson and Svensson occasionally complemented with Näslund [30–32]. Values are given according to the Swedish Gardens zone map [33] (https://zonkartan.se/).

### Genotypic data

All cultivars were genotyped with the 20 K apple Infinium^®^ single nucleotide polymorphism (SNP) array [5]. Some of the cultivars were genotyped for the current study, while others had been genotyped during previous studies [34] and ongoing research projects. Marker data was curated as described by Vanderzande & Howard et al. [17] for markers mapped to LG 3, 9, 12, and 16 on the iGLMap [9]. A subset of SNPs retained in a study on marker integration [35] with SNP calls made in Genome Studio (GS) v 2.0 (Illumina Inc.) were used. Cluster definitions were kindly made available by Howard et al. [35]. Thus, SNP calls were transformed into phased haploblock (HB) calls, which were used for downstream analysis, with each HB profile being numerically coded as 1, 2, …, *i*. Genetic positions were taken from an early draft of the iGW-map [36], and physical positions from the HFTH1 WGS [11]. Genotype calls for comparison of *NAC18.1* allele assignment were taken from Migicovsky et al. [20] and Skytte af Sätra et al. [37], assuming all accessions in those and the current study to be true-to-type.

### Phenotypic data

Date of harvest was available for individual years between 2019 and 2021 for each collection (File S1). Date of harvest was phenotyped as in ECPGR 2.1 [38]. Trees were evaluated once a week during the harvest period. Ground and cover colors were visually evaluated, as well as seed color. Starch degradation was monitored using iodine and fruits were picked (date of harvest) at iodine index of Ctifl 6–7. Growing day degrees since the last of May (i.e. 1:st of June as day 1), with base 10 °C [39], was used as phenotype for harvest date (DDH).

Flowering date was phenotyped between 2019 and 2021 as date of 50% flowering. Date of 50% flowering was phenotyped as in ECPGR 1.1 [38]. Thus, plants were evaluated three times a week during blossoming time, and flowering time was determined when approx. 50% of flowers were open, first petals started to drop. The cumulative day degrees at 50% flowering (DD_50_) were calculated relative to the last of December previous year (i.e. 1st of January as day 1), based on the daily average of the recorded temperatures. The cumulative day degrees were calculated according to [40], with the modification that the calculation was done by days, rather than by hours. For each day, the growing day degrees were calculated as

1 $$\:If\:TD{\;<\;}TU,\:{GDD}_{1}=\: \frac{F\times\left({TU}-{TB}\right)}{2}\;\times\:\:\left[1+\:\mathrm{cos}\left({\pi} + {\pi} \:\frac{\left(TD-TB\right)}{\left(TU-TB\right)}\right)\right]$$

2 $$\:If\:TD>TU,\:{GDD}_{2}=\:F\times\:\left(TU-TB\right)\times\:\:\left[1+\:\mathrm{cos}\left(\frac{\pi\:}{2}+\frac{\pi\:}{2}\:\frac{\left(TD-TU\right)}{\left(TC-TU\right)}\right)\right]$$

were GDD is the accumulation of growing day degrees during one day, TD is the average daily temperature, TB is the base temperature (4 °C), TU is the optimum temperature (25 °C), TC is the critical temperature (36 °C), F is a stress factor which here is set to 1.0, assuming no stress. In contrast to Anderson et al. [40], we did not consider the number of chill units accumulated before we started counting day-degrees, and thus added the additional requirement

3 $$\:If\:TD\;{<}\:0^{\circ}{C},{GDD}_{3}=\;{0}$$

to account for low temperatures during winter. To calculate DD_50_ the accumulated growing day degrees were then summed up.

### PRB2 data

Metabolomic data on skin concentration of PRB2 was previously described in Nybom et al. [41] and Røen et al. [42]. Briefly, fruits were collected from Swedish gene bank collections in 2002 and 2003 and Norwegian gene bank collections in 2003 (Fig. 1c, File S1). The cultivars ‘Aroma’, ‘Discovery’, ‘Ingrid Matie’, ‘Katja’, and ‘Lobo’ were common to all years and locations. Fruits [10–12] of early-ripening cultivars were harvested at physiological maturity, whereas late-ripening cultivars were stored at approximately 3–4 °C under normal atmospheric conditions until reaching comparable maturity (4–12 weeks, depending on the cultivar). To obtain a representative skin sample each fruit was cut into two halves, the more red (sunny side) and the more green (shadow side). Each half was again cut into two halves of which one was used. The fruit skin was carefully separated from the underlying flesh by peeling, immediately frozen in liquid nitrogen, then freeze-dried (subsample of approx. 15 g fresh weight) and stored at -20 °C until further analysis. The dry matter content of the skin was also determined by freeze-drying (approx. 5 g fresh weight, in duplicates). The freeze-dried skin was finely ground before extraction with 50% acidified ethanol (using 50 mM phosphoric acid). For extraction of phenolic compounds approx. 50 mg sample was used (in duplicates). The phenolic profile of the major compounds was determined by high-performance liquid chromatography (HPLC) using external standards for identification and quantification, employing methods based on those described by Tsao et al. [43] and Salminen et al. [44], with minor modifications.

### Analysis of phenotypic data

Broad sense heritability was point estimated (*Ĥ*^*2*^) using a regression approach. Briefly, Best Linear Unbiased Estimates (BLUEs) and Best Linear Unbiased Predictors (BLUPs) were calculated by fitting models with year-by-country nested within country as random effects to each phenotypic data set. The regression coefficient obtained by regressing BLUPs on BLUEs was then be used as an approximation of *Ĥ*^*2*^ [45].

For the association analysis BLUEs for each country were calculated by fitting mixed models to the observed phenotypes with genotype as fixed effect and year as random effect with random intercept using the ‘lme4’ package for R [46, 47]. For PRB2 original phenotype data was used for the association analysis, as data was only available for two years from Sweden and one year from Norway. BLUEs calculated over all observations by fitting mixed models with genotype as fixed effect and country and year as random effects, with year nested within country and random intercepts, were used for the *post-hoc* analysis and correlation analysis. Trait-trait correlations were assessed by Pearson’s product-moment correlation (*p*_*P*_) and Stuart’s tau-c (*τ*_*C*_).

### Haplotype association analysis

Here we utilized the approach for haplotype-based validation of genomic regions previously described by Skytte af Sätra [48], based on the characteristics of the 20 K SNP array [5]. Briefly, for each chromosome series of up to 30 consecutive HBs (joint HBs) were fitted as fixed effect factors to a mixed model with genotype as random effect, and a g-matrix included as a covariance matrix, using ‘lme4QTL’ and ‘AGHmatrix’ [49–51]. As each diploid cultivar has two haplotypes, the individuals in the g-matrix and the phenotypic observations were doubled, so that each individual was represented once for each haplotype. Only haplotypes up to 10 cM in length and overlapping with the prior region for validation were considered. A simple index (P_Index_) was used for evaluation of the models, defined as the product of the proportion haplotypes remaining after filtering for MAF (*≥* 0.05) and the negative log_10_-transpormed *p*-value (-log10(*p*)) for the haplotype effect. Note that here, *p*-values are used to calculate an index to identify the best candidate for *post-hoc* analysis rather than comparison with a threshold for significance testing. While model evaluation is based on the P_Index_, we also report and illustrate model *p*-values to provide a familiar scale of reference.

Subsequently, common haplotypes (MAF *≥* 0.05) with increasing or decreasing effects were identified by *post-hoc* tests. First, pairwise t-tests (*p*_*t*_) were performed between haplotypes and their average effects across individuals were estimated to identify haplotypes with increasing and decreasing effects on trait values. Second, Kolgomorov-Smirnoff tests (*p*_*ks*_) were performed to assign effects of hereto ambiguous haplotypes, and to test differences between genotypes. Dominance and interaction effects were not assessed, as they could not be estimated in a meaningful way with the current dataset.

## Results

### Heritability and trait-correlations

**Fig. 2:**
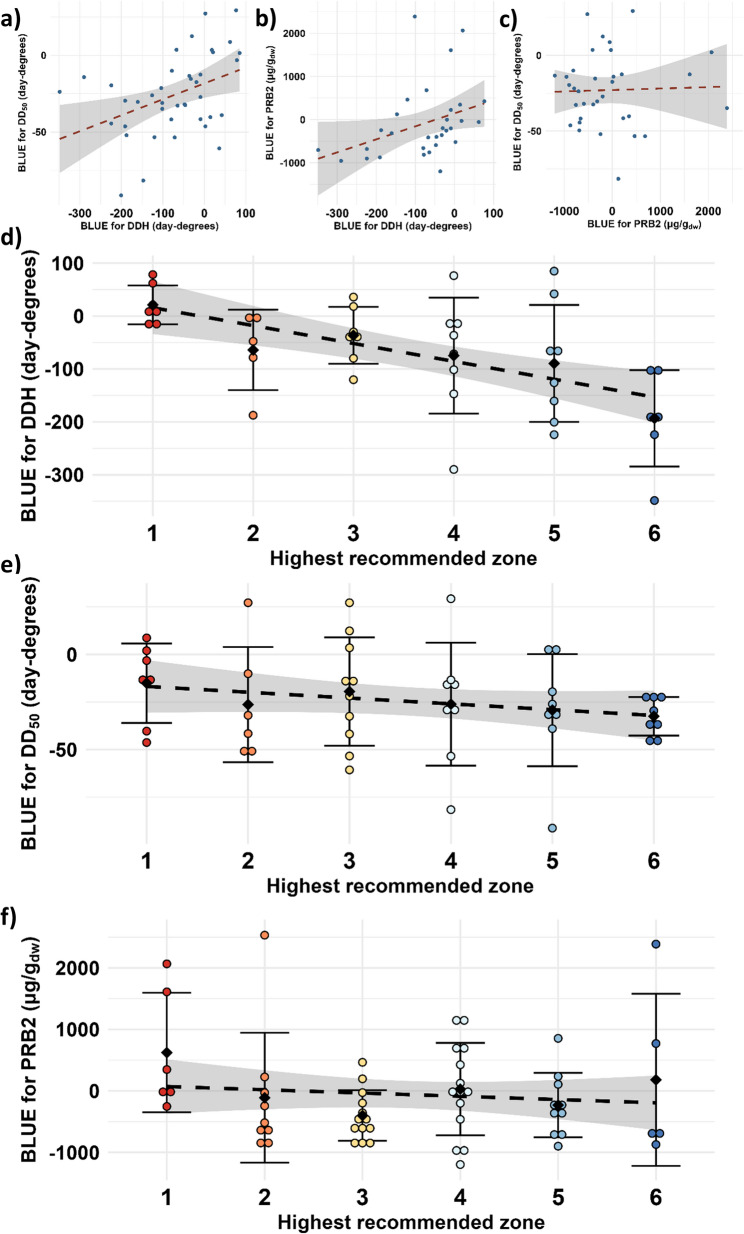
Trait-trait correlations for day degrees to harvest (DDH) and day degrees to 50% flowering (DD_50_) (**a**), DDH and fruit skin concentration of procyanidin B2 (PRB2) (**b**), PRB2 and DD_50_ (**c**), highest recommended growing-zone from pomological records (HGZ) and DDH (**d**), HGZ and DD_50_ (**e**), and HGZ and PRB2 (**f**)

All three traits, DDH, DD_50_, and PRB2, exhibited moderate to high estimates of broad sense heritability (*Ĥ*^*2*^_*DDH*_: 0.78, *Ĥ*^*2*^_*DD50*_: 0.69, *Ĥ*^*2*^_*PRB2*_: 0.61). We also found DDH to be positively correlated with both DD_50_ (*p*_*P*_ < 0.01, *r* = 0.41, Fig. 2a) and PRB2 (*p*_*P*_ < 0.05, *r* = 0.36, Fig. 2b), while DD_50_ and PRB2 were not correlated (*p*_*P*_ > > 0.1, Fig. 2c). We also found DDH to be negatively correlated with HGZ (*p*_*P*_ < 0.001, *r* = -0.56) (*τ*_*C*_: -0.48; CI_99.9%_: -0.83 – -0.12), such that cultivars recommended up to zone 6 reached harvest maturity on average 214 day-degrees earlier than cultivars recommended up to zone 1. On the other hand, DD_50_ and PRB2 were not significantly correlated with HGZ (*p*_*P*_ = 0.21 & 0.49, respectivley).

### Association analysis

**Fig. 3:**
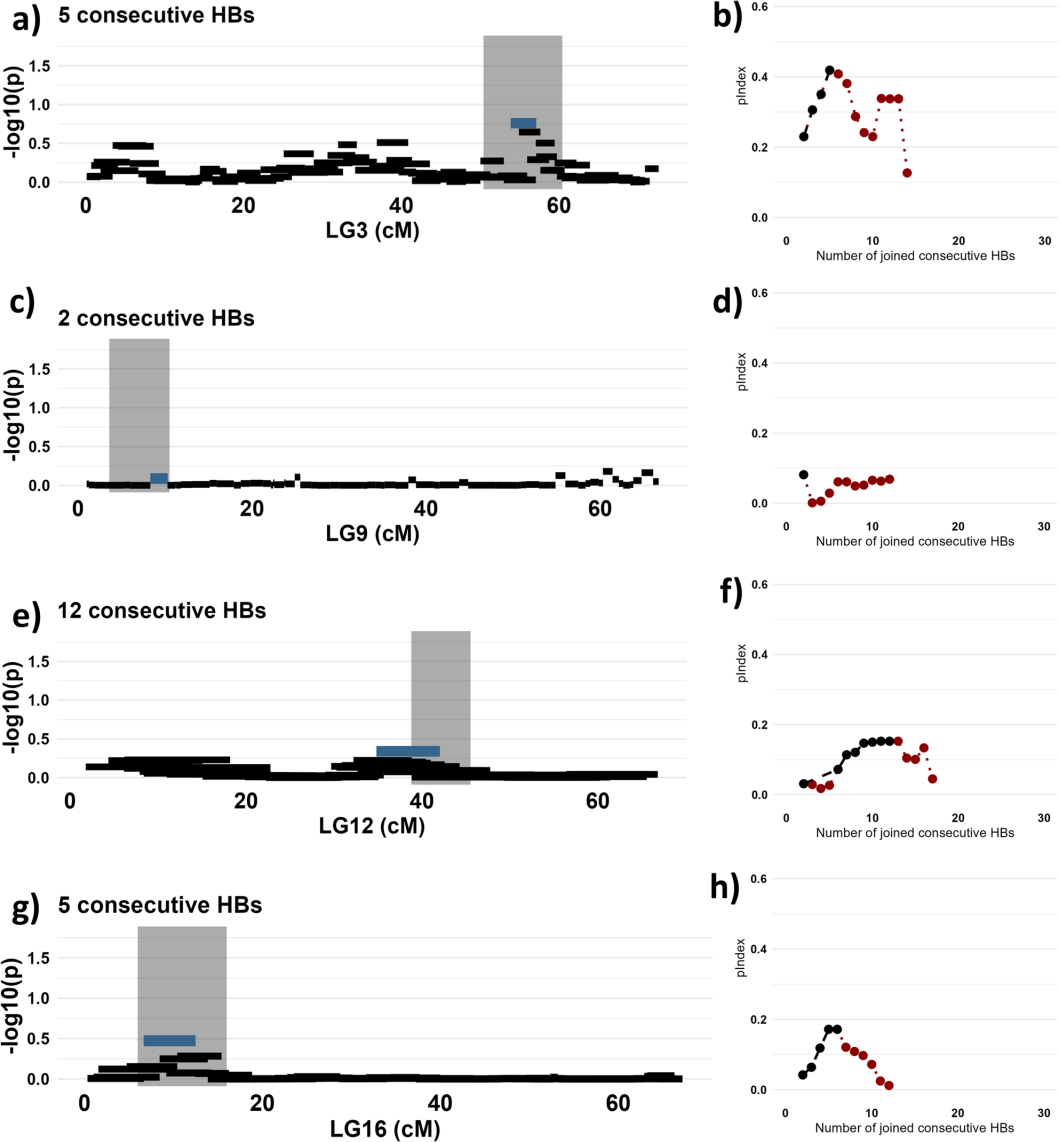
Associations by trait and linkage group (LG). **a**, **b** day degrees to harvest (DDH) and LG3, **c**, **d** day degrees to 50% flowering (DD_50_) and LG9, **e**, **f** day degrees to 50% flowering (DD_50_) and LG12, and **g**, **h** fruit skin concentration of procyanidin B2 (PRB2) and LG16. Showing (**a**, **c**, **e**, **g**) chromosome-wide pancake plots, with the –log_10_(p) of each joint HBs given on the y-axes, and the genetic position each LG on the x-axes. Each haplotype is indicated as a black horizontal bar. Approximate locations of genomic regions considered for validation are indicated by grey shaded areas. The retained haplotypes are indicated as blue bars. Also, the development of the P_Index_ (**b**, **d**, **f**, **h**) is shown, with ascending models in black and non-ascending models in red

Harvest date and the LG3 locus exhibited the strongest association in this study. A joint HB comprising five consecutive HBs, spanning *NAC18.1*, had the highest P_Index_ and a -log_10_(*p*)-value of 0.76 (Fig. 3a, b). Additional peaks occurred on other parts of the chromosome, such that the haplotype with the highest P_Index_ ranged from 12 to 29 cM and had a -log_10_(*p*)-value of 3.8 (not shown).

We observed a very weak association between flowering time and the LG9 locus. The haplotype with the highest P_Index_ occurred already for 2 consecutive HBs and had a -log_10_(*p*)-value of only 0.09 (Fig. 3c, d). However, all haplotype models with more than 8 consecutive HBs indicated a clear increase in P_Index_ around ~ 30 cM, peaking with 9 consecutive HBs spanning 25.5 to 34.8 cM and having a -log_10_(*p*)-value of 1.3 (not shown).

While relatively weak, the LG12 locus exhibited a stronger association with flowering time than the LG9 locus, such that a haplotype comprising 12 consecutive HBs had the highest P_Index_ and a -log_10_(*p*)-value of 0.34 (Fig. 3e, f). At the distal part of the chromosome a region around 10 cM exhibited increased P_Index_ across models, peaking with 8 consecutive HBs spanning 4–11 cM and having a -log_10_(*p*)-value of 0.55 (not shown).

The LG16 locus showed a moderate association with PRB2 concentration, such that the haplotype with the highest P_Index_ while overlapping with the posterior defined interval had a -log_10_(*p*)-value of 0.47 for 5 consecutive HBs (Fig. 3g, h). Longer haplotypes overlapping with the targeted genomic region exhibited -log_10_(*p*)-values of up to 1.43, but these exceeded the size threshold and/or had a very low proportion individuals retained after frequency filtering.

### *Post-hoc* analysis

**Fig. 4:**
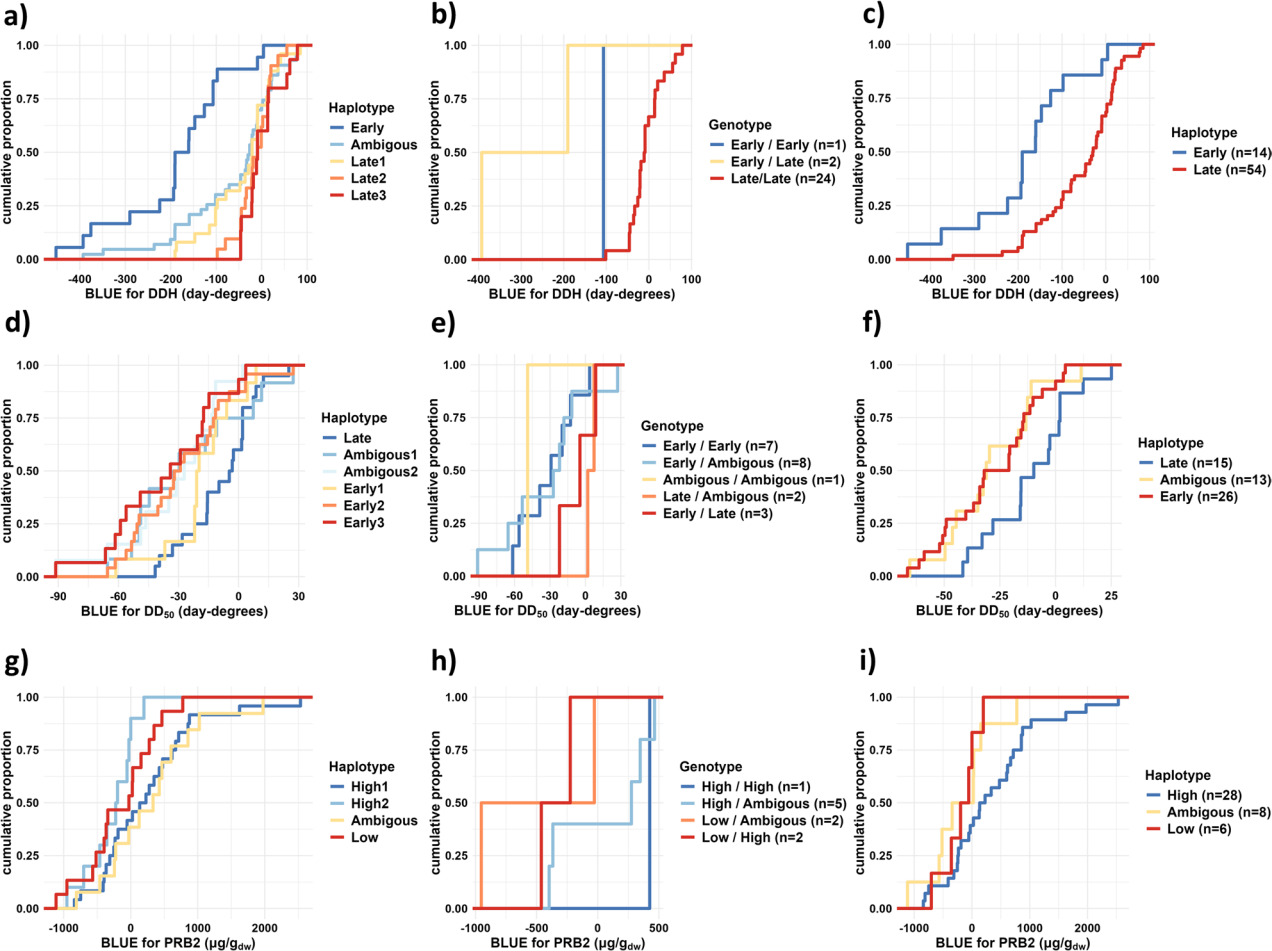
Phenotypic distribution of haplotypes and genotypes for day degrees to harvest (DDH) and linkage group (LG) 3 (**a**, **b**, **c**), day degrees to 50% flowering (DD_50_) and LG12 (**d**, **e**, **f**), fruit skin concentration of procyanidin B2 (PRB2) and LG16 (**g**, **h**, **i**) indicating initially assigned haplotypes (**a**, **d**, **g**), full effective genotypes (**b**, **e**, **h**), and cultivars with only one assigned effective haplotype (**c**, **f**, **i**)

For the LG3 locus for harvest date there were five common haplotypes. One haplotype was significantly different from the others (*p*_*t*_ < 10^− 6^), had the lowest mean for DDH, and was designated as *Early* (Supplementary Table 2). Three of the haplotypes were not statistically different from one another (*p*_*t*_ > 0.05), designated as *Late1-3*, while the effect of the fifth haplotype was initially designated *Ambiguous* as it was associated with slightly earlier ripening than the latest haplotypes (0.05 > *p*_*t*_ > 0.01) (Fig. 4a). A second evaluation of cultivars with two assigned haplotypes revealed cultivars assigned as homozygous *Late* to be indistinguishable from individuals with one *Late* allele and one *Ambiguous* allele (*p*_*ks*_ = 0.15). Consequently, the initial *Ambiguous* allele was considered to have the same effect as a *Late* allele. Thus, 24 cultivars were assigned homozygous for the *Late* alleles and two cultivars were assigned heterozygous *Early*/*Late* with an average adjusted mean for DDH 284.8 day-degrees lower than the homozygous *Late*. A single cultivar, ‘Snygg’, was homozygous *Early* (Fig. 4b). Considering cultivars with an assigned effect of only one haplotype, individuals carrying one *Early* allele had average adjusted means for DDH 132.1 day-degrees lower than individuals carrying one *Late* allele (Fig. 4c). We found no inconsistencies with assignment of genotypes in relation to Skytte af Sätra et al. (2024) or Migicovsky et al. (2021). Additionally, the *Early* allele was found to be the minor allele (Allele Frequency, AF = 0.15) across all 122 haplotype samples retained for *post-hoc* analysis.

Regarding the four common haplotypes at the LG9 locus investigated for potential association with flowering date, only two differed significantly (*p*_*t*_ < 0.01). However, none of the diploid genotypes (*n* = 100) showed significant differences (*p*_*ks*_ > > 0.1 ) and diploid genotypes without *Ambiguous* alleles were very rare (n *≤* 3). Thus, under these conditions, the effect of the LG9 locus could not be verified in this germplasm.

For the LG12 locus for flowering date, the germplasm comprised five common haplotypes. Three haplotypes were associated with significantly (*p*_*t*_ < 0.01) lower DD_50_ than one of the haplotypes, designated *Late* and *Early1-3*, respectively (Fig. 4d, Supplementary Table 3). The two other haplotypes had ambiguous effects. Thus, 21 cultivars had assigned genotypes at the LG12 locus (Fig. 4e). No significant differences were found between genotypes (*p*_*ks*_ > 0.05), though the heterozygote *Early*/*Late* genotype was represented by only three cultivars. Cultivars carrying two *Early* alleles reached full bloom on average 24.6 day-degrees earlier than individuals being heterozygous *Early*/*Late*. For individuals with only one assigned effective haplotype, neither the *Early* or the *Ambiguous* alleles were significantly different from the *Late* haplotype (*p*_*ks*_ = 0.08), though individuals having the *Early* or the *Ambiguous* allele flowered in average 18 day-degrees earlier than individuals having the *Late* allele (Fig. 4f). We also note that the *Late* allele was the minor allele, with an AF of 0.21 across all 96 haplotype samples, compared to the more common *Early* alleles (AF = 0.53).

Considering the association between the LG16 locus and content of PRB2, the germplasm comprised four common haplotypes. Two haplotypes were associated with significantly (*p*_*t*_ < 0.05) higher PRB2 concentration than a third haplotype, designated *High1*, *High2*, and *Low* alleles, respectively (Supplementary Table 4). The fourth haplotype was not significantly different from the other three and was designated *Ambiguous* (Fig. 4g). Only 10 cultivars had assigned diploid genotypes, so pairwise differences were not significant (*p*_*ks*_ > 0.5), though individuals that were homozygous *High*/*High* had on average 768 µg/g_dw_ higher PRB2 than those that were heterozygous *Low*/*High* (Fig. 4h). For individuals with only one assigned allele, neither the *Low* nor the *Ambiguous* alleles were significantly different from the *High* haplotype (*p*_*ks*_ > 0.1). On the other hand, individuals with a *Low* or *Ambiguous* allele had on average 526 and 535 µg/g_dw_ lower PRB2 than individuals with a *High* allele (Fig. 4i). Lastly, the *Low* allele is rare (MAF = 0.16) across the 62 individuals, and the *High* allele appears to be common (AF = 0.60).

## Discussion

### Harvest date

The timing of phenological growth stages is expected to be critical to adaptation to different local temperate climates along a continental – boreal gradient. We found harvest date expressed in day-degrees to be significantly correlated with historical adaptation to Swedish climate, indicative of directional selection for the trait. Using phased 20 K SNP array data we were able to validate the genomic region around previously described *NAC18.1* locus for harvest date on LG3, verifying the effect of the locus and demonstrating the validity of the approach. In previous work the allele associated with late harvest was found to be minor (MAF = 0.33) in a Swedish diversity germplasm [22] while we here find the opposite (MAF = 0.15). This is most likely due to the skew towards a modern breeding germplasm in the current study, resulting in a stronger influence of the western European apple gene pool [34, 52]. The role of harvest date in climate adaptation is also supported by previous work, which has provided further support for directional selection on the *NAC18.1* locus among apple cultivars adapted to a boreal climate [37]. On the other hand, no indication of directional selection has been found among apple cultivars adapted to continental climate, suggesting that the allele frequency in this ancestral group reflects a state of migration-mutation-drift equilibrium in the absence of selection. However, it should be noted that the number of cultivars used in the current analysis is relatively small (*n* = 111), and that only a single cultivar overlapped across the three locations used for phenotyping (Fig. 1).

Despite the moderate number of cultivars, DDH had the highest estimated broad sense heritability in this study. The germplasm analyzed in Sweden and Norway had similar allele frequencies for the LG3 haplotypes, while the *Early* allele had a much higher frequency in the material from Finland (AF = 0.13, 0.05, and 0.47 in Sweden, Norway, and Finland, respectively) where two of the *Late* alleles were also missing (Supplementary Table 5). With such imbalanced data, Genotype × Environment interactions and Environmental effects will be confounded with Genotype effects such that the effect of the *Early* allele might in part be because it is common in the Finnish collection. On the other hand, such difference in allele frequency would be expected as most of Finland has a boreal climate which would favor the allele for early harvest among local cultivars, in accordance with the significant correlation between DDH and HGZ. Differences in local climates are also accounted for, to some degree, by using day degrees to harvest, rather than calendar date.

### Flowering date

In contrast to harvest date, the time to full bloom expressed in day-degrees was not correlated with historical climate adaptation. This could indicate that flowering time has not been a critical trait for historical adaptation to different Nordic climates, or that meaningful variation for selection to act on has been largely lacking. An effect of the previously described LG9 locus could not be validated [23]. A locus for chilling requirement has previously been mapped to the same region [53], thus one possible explanation for this is that this locus is involved in a QTL × Environment interaction such that the effect diminishes under Nordic conditions where chilling requirement is not expected to be a limiting factor for time of bud break. Another explanation could be a lack of variability at the LG9 locus in the current study. For example, in Allard et al. [23], the LG9 locus segregated only in the cultivars ‘Granny Smith’ and ‘Belrene’, both of which to our knowledge lack pedigree connections to our data set. Urrestarazu et al. [22] identified a genomic region at the top of Chr9 associated with time of bud break. However, the strongest association was found for two loci in strong linkage disequilibrium (LD) in the ‘Western’ subpopulation, but not in the ‘Northern European’ subpopulation. At both loci, the minor allele frequency was much lower in the ‘Northern European’ subpopulation, such that one homozygous class was represented by a single cultivar. This indicates that the reported association between flowering time and a genomic region at the proximal part of Chr9 is caused by a haplotype that exists at a relatively high frequency only in the ‘Western’ apple subpopulation. Thus, this locus would be expected to be less variable in the current Nordic germplasm. We also note that the central peak in association signal between haplotype and flowering time coincides with a predicted gene with annotated similarity to PHYC (encoding phytochrome C) [54]. In apple, Chr9 is syntenic to chromosome 17 (Chr17), and a QTL for autumn phenology has recently been mapped to Chr17, also coinciding with a putative PHYC homolog [48].

On the other hand, we did verify the effect of variable haplotypes coinciding with the previously described LG12 locus for bud break. However, some cultivars included in the current study were parents or ancestors of the multi-parental populations (MPP) used by Allard et al. [23], and genotype assignments were not fully consistent. The cultivars ‘Cox’s Orange Pippin’, ‘Golden Delicious’, ‘Jonathan’, ‘Delicious’, and ‘Prima’ were all assigned homozygous for the allele for late flowering at the LG12 locus in Allard et al. [23], while we have assigned ‘Cox’s Orange Pippin’, ‘Golden Delicious’, and ‘Jonathan’ to carry one *Early* allele each. There are several possible explanations for this. First of all, the only cultivar that is common between the studies and a direct parent of one of the segregating populations in Allard et al. [23] is ‘Delicious’. The other common cultivars are ancestors and as Allard et al. [23] do not describe the transmission of the LG12 QTL haplotypes it might be that the QTL genotypes are partially inferred without direct segregation supporting their effects. The approach used by Allard et al. [23] also assumes loci with only two different effects, *Q*/*q*, and the only cultivar assigned a *q* allele is ‘Granny Smith’. Assuming that the *q* allele of ‘Granny Smith’ is absent in our germplasm, we would distinguish effects of alleles with smaller effects which would have been assigned the same effect in relation to the *q* allele of ‘Granny Smith’ in Allard et al. [23]. Thus, while the allele associated with late flowering was clearly major in Allard et al. [23], the *Late* allele in the current Nordic breeding germplasm is rare, existing predominantly among descendants of ‘Golden Delicious’. It is also worth noting that the phenotypic data used by Allard et al. [23] was collected under ‘mediterranean’ – ‘lusitanian’ climates while the data for the current study was collected under ‘continental’ – ‘nemoral’ – ‘atlantic north’ climates [55], such that differences between studies could partly be attributed to Genotype × Environment interactions. Lastly, we note that Urrestarazu et al. [22] report a genomic region around 14.5 Mb of Chr12 of the GDDH13v1.1 WGS to be associated with flowering time in one study location. However, we did not account for this locus as a BLAST of the SNP probe sequence (not shown) raised doubts abouts its position and relevance for the current genomic region.

In the three traits reported here, data was available for the largest number of individuals for DD_50_ (*n* = 189), which was also the trait with the largest number of cultivars in common between the three locations (Fig. 1). Despite this, DD_50_ had a lower estimated broad sense heritability than DDH. Alleles associated with late and early flowering had similar frequencies across the three germplasm collections, in accordance with the lack of correlation between DD_50_ and HGZ, though *Early3* had a higher frequency in the Finnish collection than in the Swedish and Norwegian collections (Supplementary Table 5). The *Early1* allele was also absent in the Finnish collection. However, this might reflect a random sampling error as the Finnish collection was the smallest phenotyped for this trait (*n* = 53) with only 15 cultivars having at least one haplotype with an estimated effect.

### Polyphenols

We also validated effects of common contrasting haplotypes around the *LAR1* locus on LG16 for the fruit peel concentration of the polyphenol PRB2. Khan et al. [27] previously reported dihybrid segregation at the *LAR1* locus in a ‘Fiesta’ × ‘Prima’ full-sibling population with procyanidin dimer II concentration as phenotype. In these two cultivars, we only identified an effect of one of the haplotypes, a *High* allele in ‘Prima’. Leforestier et al. [56] identified a SNP around the *LAR1* locus associated with differentiation between sweet and bitter cultivars. In our study, that SNP (20 K index: 647) distinguished the *Ambiguous* haplotype from the *High* and *Low* haplotypes. We note that the *Low* allele appears to be relatively rare in the current germplasm, being predominantly present in cultivars from the Swedish breeding programme and their close relatives. This indicates that combining local adaptation with high PRB2 concentration, for e.g. juice or cider production, would be feasible in the available germplasm, especially since the increasing allele has been reported to be dominant [27], but requires dedicated breeding. Another option for breeding new cultivars for juice and cider production would be to make crosses with traditional cider cultivars. However, the *LAR1* locus might be of limited value in such setting, as neither QTL-mapping [26] nor GWAS [57] have revealed any association between the *LAR1* locus and concentration of phenolic compounds in cider apples, presumably because of low variability in the cider germplasm. Breeding for low PRB2 concentration combined with phenology adaptation would also be feasible, but would be more constrained in choice of parents and require an efficient MAS platform, because of low frequency of the *Low* allele in the current germplasm and the reported dominance of the *High* allele [27]. The *LAR1* locus has been targeted by platforms designed for MAS, but have been assessed for their effect on crispness and firmness rather than concentration of fruit polyphenols and exhibits inconsistencies between germplasms [25].

Among the three traits investigated in this study, phenotypic data was available for the smallest number of cultivars for PRB2 (*n* = 88). Such sample size would be considered insufficient for traditional GWAS, and here led to a small number of phenotyped cultivars having assigned genotypes for *post-hoc* analysis. We found no major differences in allele frequencies between the Swedish and the Norwegian collections, as expected from the lack of correlation between PRB2 and HGZ.

### Concluding remarks

The results presented in this study provide valuable information for applications in the Nordic breeding programs. The previously described genomic region on LG3 associated with harvest date had a significant effect also under Nordic conditions. While the current study provides information that can be of some use for MAPS, genotyping with the previously described single SNPs would increase the informativeness of the data. Once validated, these single SNPs could also be useful for deployment in MASS. However, as the LG3 locus associated with harvest date has been reported to affect fruit firmness and storability, future work is needed to study to which degree other loci can counter the undesirable effects of the LG3 locus. Considering flowering, the LG9 locus from previous studies does not seem to have an effect under Nordic conditions. However, descendants of ‘Golden Delicious’ could be important donors of the *Late* allele at the LG12 locus for Nordic apple breeding, though these are mostly foreign cultivars with poor local adaptation. Thus, breeding for this combination might cause a conflict between late flowering and traits associated with adaptation to e.g. middle or northern Sweden. Thus, future research and breeding efforts would benefit from a better dissection of the genetic control of flowering time in a relevant germplasm. Selection for *Low* or *High* alleles at the *LAR1* locus seems feasible, but efficient MASS would require development of better markers.

## Supplementary Information

Supplementary Material 1.

## Data Availability

BLUEs for HHD, DD_50_, and PRB2, haplotype genotypes, and SNP-profiles of the common haplotypes are available in Supplementary Tables 1 - 4. GenBIS IDs for accessions from the Finnish gene bank at LUKE are given in Supplementary Table 6.
